# Transcranial Pulse Stimulation with Ultrasound in Alzheimer's Disease—A New Navigated Focal Brain Therapy

**DOI:** 10.1002/advs.201902583

**Published:** 2019-12-23

**Authors:** Roland Beisteiner, Eva Matt, Christina Fan, Heike Baldysiak, Marleen Schönfeld, Tabea Philippi Novak, Ahmad Amini, Tuna Aslan, Raphael Reinecke, Johann Lehrner, Alexandra Weber, Ulrike Reime, Cédric Goldenstedt, Ernst Marlinghaus, Mark Hallett, Henning Lohse‐Busch

**Affiliations:** ^1^ Department of Neurology Laboratory for Functional Brain Diagnostics and Therapy High Field MR Center Medical University of Vienna Spitalgasse 23 Vienna 1090 Austria; ^2^ Applied Research Center Storz Medical AG Lohstampfestrasse 8 Tägerwilen 8274 Switzerland; ^3^ Rheintalklinik Outpatient Department Manual Medicine Center for Movement Disorders Thürachstraße 10 Bad Krozingen 79189 Germany; ^4^ Human Motor Control Section NINDS NIH 10 Center Drive Bethesda MD 20892‐1428 USA

**Keywords:** Alzheimer's disease, brain stimulation, ultrasound

## Abstract

Ultrasound‐based brain stimulation techniques may become a powerful new technique to modulate the human brain in a focal and targeted manner. However, for clinical brain stimulation no certified systems exist and the current techniques have to be further developed. Here, a clinical sonication technique is introduced, based on single ultrashort ultrasound pulses (transcranial pulse stimulation, TPS) which markedly differs from existing focused ultrasound techniques. In addition, a first clinical study using ultrasound brain stimulation and first observations of long term effects are presented. Comprehensive feasibility, safety, and efficacy data are provided. They consist of simulation data, laboratory measurements with rat and human skulls and brains, in vivo modulations of somatosensory evoked potentials (SEP) in healthy subjects (sham controlled) and clinical pilot data in 35 patients with Alzheimer's disease acquired in a multicenter setting (including neuropsychological scores and functional magnetic resonance imaging (fMRI)). Preclinical results show large safety margins and dose dependent neuromodulation. Patient investigations reveal high treatment tolerability and no major side effects. Neuropsychological scores improve significantly after TPS treatment and improvement lasts up to three months and correlates with an upregulation of the memory network (fMRI data). The results encourage broad neuroscientific application and translation of the method to clinical therapy and randomized sham‐controlled clinical studies.

## Graphical abstract

A new clinical brain stimulation technique is introduced, based on single ultrashort ultrasound pulses (transcranial pulse stimulation, TPS). Comprehensive feasibility, safety, and efficacy data for this new therapy are provided. Two weeks TPS treatment of 35 Alzheimer's patients reveals high treatment tolerability, no major side effects, and improved memory performance lasting up to three months.

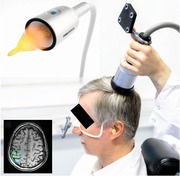

## 1 Introduction

**Figure 1:**
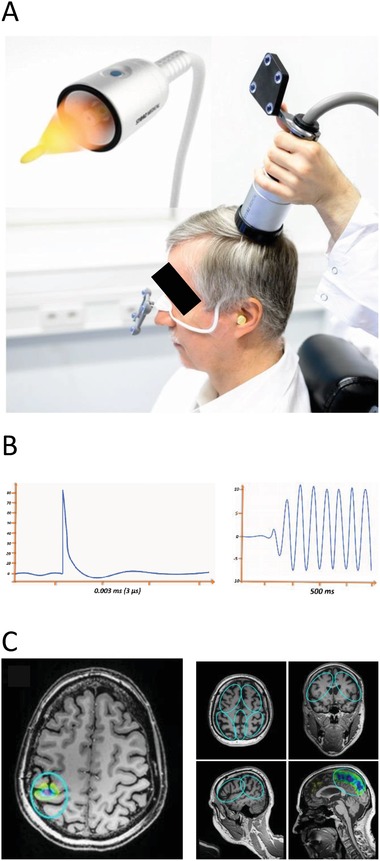
Transcranial Pulse Stimulation technique TPS. A) Goggles (affixed to the head) and TPS handpiece are equipped with infrared reflectors for visualization and tracking of the TPS focus to regions of interest in individual anatomical MR images. For TPS treatment plenty of bubble‐free ultrasound gel has to be applied on skin and hair at the treatment area to avoid acoustic impedance borders. B) TPS applies ultrashort ultrasound pulses lasting about 3 µs (left) which reduce the risk for brain heating or secondary maxima. Previous brain sonication techniques typically use ultrasound trains in the range of several hundred ms (right). C) Reliable focal targeting of TPS either to a single stimulation focus (left) or for targeting region of interest borders (blue circles, right). Each pulse leaves a colored mark inside the ROI with colors indicating local pulse counts (yellow to pink = low to high numbers). Consent for publishing this figure exists.

Recently, several publications have reported the potential of ultrasound to stimulate the human brain in a highly focal and precisely targeted manner.^1^, ^2^ However, in contrast to ultrasound tissue ablation,^3^ for clinical brain stimulation no certified systems and clinical data exist and the current techniques have to be further developed. Here we introduce a new ultrasound sonication technique, which was specifically developed for clinical applications and is based on single ultrashort ultrasound pulses (3 µs) repeated every 200–300 ms (transcranial pulse stimulation (TPS), **Figure** **1**). In a comprehensive approach, we provide preclinical and clinical feasibility, safety, and efficacy data for TPS. Clinical brain stimulation alternatives to existing electrophysiological brain stimulation techniques are urgently needed, since current techniques suffer from limited targeting due to conductivity effects and lack of deep stimulation capabilities.^4^, ^5^, ^6^ Ultrasound can be reliably targeted and is the first technique that allows noninvasive deep brain stimulation.^7^ Here we demonstrate, that the target for the new TPS technique can be spatially distinct, highly focal, and is not restricted to superficial layers of the brain. For brain therapy, this enables a controlled modulation of a specific brain region without unwanted costimulations of other brain areas. Clearly defining which brain areas are affected by the stimulation and which are not, is an important advance for clinical application and neuroscientific research.

Experimental preclinical studies have already shown that noninvasive focal ultrasound applications may modulate the function of healthy human brains.^8^, ^9^ Effects were demonstrated for milliseconds or seconds and up to one hour in macaque monkeys.^10^ Initial clinical data concerning nontargeted stimulation and a case report are also available.^11^, ^12^ TPS extends current literature by providing a new navigated stimulation technique which is clinically certified (CE mark). It consists of a mobile single transducer system which avoids long sonication trains,^2^, ^13^ and therefore the risks of secondary stimulation maxima or brain heating can be avoided.^14^, ^15^

We also present the first patient study (Alzheimer's disease, AD) and first investigation of long‐term effects (up to three months) for ultrasound brain stimulation. In more detail, the feasibility of TPS for targeted and focal energy deposition was tested by simulations and laboratory measurements with rat and human skulls as well as brain specimens. Safety, feasibility, and efficacy of brain stimulation was then investigated by in vivo rat experiments and in vivo modulations of somatosensory evoked potentials (SEP) in healthy human subjects (sham controlled). Finally, preliminary clinical efficiency was investigated by an uncontrolled pilot study in 35 patients with probable AD using CERAD^16^ neuropsychological scores and high field functional magnetic resonance imaging (fMRI) data. Since a major clinical advantage of TPS is the potential to act as an independent add‐on therapy, we included patients with ongoing and optimized standard clinical treatment.

## 2 Results

### 2.1 TPS Technique

The newly developed TPS system consists of a mobile single transducer and an infrared camera system (Polaris Vicra System by Northern Digital Inc.) for MR based neuronavigation (NEUROLITH, Storz Medical AG, Tägerwilen, Switzerland, Figure 1). The camera tracks the positions of the handpiece and the head of the patient via goggles affixed with infrared markers. For TPS treatment plenty of bubble‐free ultrasound gel has to be applied on skin and hair at the treatment area to avoid acoustic impedance borders.

To standardize treatments for all patients by means of treatment visualization and recording, the system allows defining standardized target volumes of interest for each individual participant's MRI (ellipsoid regions of interest (ROIs), Figure 1C). This individual real time tracking enables standardized focal brain stimulation over the whole study population with adequate movements of the handpiece over the skull. Additionally, tracking of stimulation pulses is possible with each pulse leaving a colored mark in the visualization. TPS generates single ultrashort (3 µs) ultrasound pulses with typical energy levels of 0.2–0.3 mJ mm^−2^ and pulse frequencies of 1–5 Hz (pulses per second). Based on unpublished experimental series for dose finding and CE approval, limitations of the system have been set to the following values: maximum energy flux density: 0.25 mJ mm^−2^ at 4 Hz, maximum spatial‐peak‐temporal‐average intensity *I*_SPTA_: 0.1 W cm^−2^ (fulfilling DIN EN 61689), maximum number of pulses per treatment: 6000, maximum peak pressure 25 MPa. Below 40 MPa no tissue lesions have yet been reported.

Sonication of target areas is done via variable stand offs at the handpiece for depth regulation and manual movement of the handpiece over the skull with real time pulse tracking. For highly focal applications (Figure 1C left), the handpiece may be fixed at a constant position over the skull. The whole treatment session can be recorded for post hoc evaluation of the individual intracerebral pulse localizations.

TPS differs from current brain sonication concepts with focused ultrasound (FUS^2^) since TPS applies single ultrasound pulses and no periodic waves. With periodic waves and long sonication trains, there is a danger of brain heating.^14^ A further problem concerns the danger for generation of unintended secondary stimulation maxima. They may occur due to interaction of reflections or generation of standing waves. These drawbacks of FUS cannot occur with the current settings of the TPS system. Another expected advantage of TPS is better skull penetration due to a dominance of lower frequencies within the TPS pulse. In contrast to many FUS systems, TPS uses an electromagnetic pulse generator with advantages for pulse stability.

### 2.2 Evidence for TPS Focal Energy Transmission

The experimental setting for the laboratory measurements is shown in Figure S1 in the Supporting Information. The investigations demonstrate that TPS can generate a focal stimulation pulse below the skull (Figure S2, Supporting Information).

#### 2.2.1 TPS Data Simulations

3D simulation of temporal peak intensities showed that a highly focal energy pulse can be generated through the skull. Calculations for two human skulls showed a consistent peak intensity drop (skull attenuation) of about 65% at spatial peak. Such values are important for defining adequate energy levels for clinical therapy.

#### 2.2.2 Human Skull and Brain Sample Measurements

Measurements of temporal peak intensities with two human skulls and ten human brain samples confirmed the transmission of a focal energy pulse without occurrence of secondary maxima. However, compared to free degassed water, the human skulls produced a temporal‐peak intensity drop of 80–90% and a slightly widened and shifted focus (Figure S2B, Supporting Information). For human brain tissue we found a considerable variability of results depending on the state of the post mortem tissue and the suspected amount of decay gases (brains were 0–7 d post mortem). With consideration of tissue state, overall results corresponded to published values for sound wave attenuation in human brain tissue, which is in the range of 0.58 dB cm^−1^ MHz^−1^.^17^

#### 2.2.3 Rat Skull Measurements

Rat skull measurements (Figure S2C, Supporting Information) again confirmed a focal energy pulse. Rat skulls produced a mean pressure drop of about 29%. Depending on pulse energy, the detailed losses were: 20.3% for 0.1 mJ mm^−2^, 28.8% for 0.35 mJ mm^−2^ and 37.3% for 0.55 mJ mm^−2^. This illustrates the importance of comparative measurements when trying to translate animal data from new ultrasound techniques to human applications.

### 2.3 Evidence for TPS Safety and Neuromodulatory Efficacy

#### 2.3.1 TPS Safety Investigations in Anesthetized Rats

Brain preparations of 80 rats treated with a constant TPS focus with varying energy levels did not show any intracranial, subarachnoid, or subdural bleeding in any rat of any group. Histological investigations of two brains per group did not show any abnormalities and no indications for blood brain barrier damages in any TPS group. A second study tested various energy doses in five rats (15‐, 150‐, and 300‐fold energy levels compared to the human doses allowed with the certified TPS system). Anatomical in vivo MRI did not show any brain damage up to 150‐fold energy levels (Figure S3, Supporting Information). Despite considerably lower pressure wave attenuation by rat skulls (29% in rat instead of 85% in human skulls), TPS application in living rats was safe—even at much higher energy levels than those used for the clinical study.

#### 2.3.2 TPS Safety and Neuromodulatory Efficacy in Healthy Subjects

**Figure 2:**
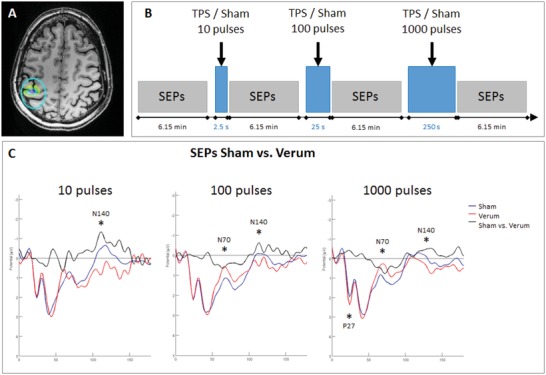
TPS neuromodulation in ten healthy subjects. A) Transcranial pulse stimulation (TPS): Highly focal stimulation of the primary somatosensory representation of the right hand, definied on individual MR images. B) Study design: sham and verum stimulation were compared within the same subjects. 1 baseline measurement of median nerve somatosensory evoked potentials (SEPs) was followed by three runs of TPS stimulation (10/100/1000 pulses in fixed order), each followed by an additional recording of SEPs. C) SEP results: Increase of TPS neuromodulation effects with an increase of pulses: with ten pulses N140 decreased (verum stimulation in red), with 100 pulses additionally N70 increased and with 1000 pulses also P27 decreased.

In ten healthy subjects, safety and efficacy of TPS for modulation of human neuronal activity was investigated using median nerve SEPs (**Figure** **2**). When targeting the primary somatosensory cortex and comparing sham with verum stimulation, clear modulations of evoked brain activity were found. All stimulations were well tolerated. The factorial analysis showed an increase of neuromodulatory effects with an increase of TPS pulses. With ten pulses only 1 SEP component was affected, with 1000 pulses three SEP components were affected. This indicates a dependence of TPS efficacy on total energy integrated over time.

### 2.4 Preliminary Evidence for TPS Clinical Efficacy and Safety

35 patients from two clinical centers with probable AD and continuous state of the art treatment were treated with TPS for 2–4 weeks. Center 1 (Vienna, Austria, lead) used a navigated approach to target AD relevant regions of interest. Outcome was compared with a non‐navigated global brain stimulation approach at center 2 (Bad Krozingen, Germany) as previously used in animal studies.^18^

#### 2.4.1 Patient Safety Evaluations

At both centers, patient evaluations during a three month follow up period (clinical examinations, patient reports, MRI) did not show any relevant side effects. A detailed quantification performed by center 1 resulted in 4% headache (headache history partly present), 3% mood deterioration, and 93% none. Visual analogue scale evaluation (VAS 0–10) of within‐treatment pain or pressure experience resulted in 92% VAS 0, 7% 1–5, 1% 6–8 (pain) and 83% 0, 15% 1–5, 2% 6–8 (pressure).

Evaluation of anatomical MRIs including T2* and FLASH images before and after stimulation did not reveal any hemorrhages, edema, or any other type of new intracranial pathology.

#### 2.4.2 Neuropsychological Improvements in AD

**Figure 3:**
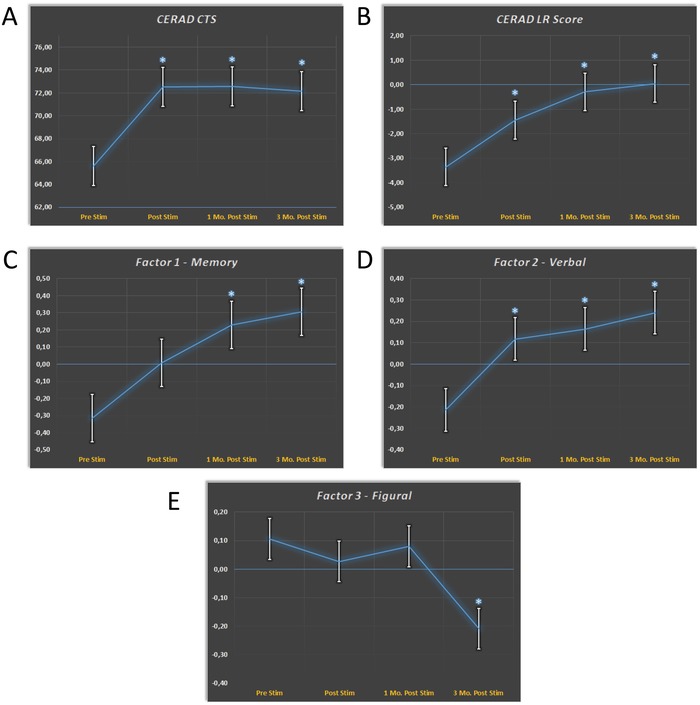
Neuropsychological improvements in Alzheimer's disease after TPS therapy. CERAD score changes (mean +/− 1 standard error) over time. Significant score changes relative to the baseline Prestim are marked by an asterisk. A) The global outcome parameter for the patients cognitive state CERAD CTS (corrected total score) improves significantly after TPS treatment. B) The CERAD logistic regression score (LR) also improved. LR focuses on tests important for AD type dementia. Results for CERAD Factors are shown in (C)–(E). Whereas C) Memory and D) Verbal functions improved over time, E) Figural functions declined. This can be explained by the fact that occipito‐parietal areas involved in figural processing, were not included in the treatment scheme.

The CERAD corrected total score (CTS) as the major global outcome parameter for the patients' cognitive state improved significantly after treatment and remained stable over three months (**Figure** **3**A). The CERAD logistic regression score (LR) focuses on tests important for AD type dementia and also improved significantly after treatment with stability over three months (Figure 3B). The CERAD principle component analysis (PCA) allows separate monitoring of the cognitive components for memory (MEMORY), verbal processing (VERBAL), and visuospatial processing (FIGURAL). Here, an interesting dissociation was found. Whereas MEMORY and VERBAL improved significantly after treatment, FIGURAL performance declined (Figure 3C–E). The FIGURAL decline was related to study center 1, which—in contrast to center 2—did not stimulate the occipito‐parietal cortex, an important area for visuospatial processing. This finding supports a specific treatment effect only found for stimulated networks. Corresponding to the CERAD results, subjective evaluation of memory performance (SEG scale) also improved significantly. Importantly, analysis of depression scores showed that neuropsychological improvements were not driven by changes of depressive symptoms. Detailed results can be found in the Supporting Information.

#### 2.4.3 Supporting Functional MRI Results

FMRI investigations in subgroups of patients confirmed a specific upregulation of the memory network after TPS therapy (Figure S4, Supporting Information). FMRI resting state data (*N* = 18) showed increased functional connectivity for hippocampus, parahippocampal cortex, parietal cortex, and precuneus. Increased functional connectivity values were significantly correlated with the CERAD scores (Table S1, Supporting Information) indicating that upregulation of the memory network is related to cognitive performance. FMRI task data (*N* = 9), achieved with a widely used memory test,^19^ confirmed a specific activation increase in bilateral hippocampus after TPS.

## 3 Discussion and Conclusion

The application of ultrasound for brain therapy has become a hot topic as it bears the potential for providing a new class of semi‐invasive (ablation^20^, ^21^, ^22^ or blood brain barrier opening^23^), or noninvasive (brain stimulation) brain therapies. However, for clinical brain stimulation the current techniques have to be further developed, and certified systems are required. Promising clinical applications particularly concern neurodegenerative disorders. Alzheimer's and Parkinson's disease are among the most important medical problems within our ageing society. Available treatments are limited, and such patients are therefore major candidates for clinical benefits of new add‐on therapies.^24^ In this article, we describe a new brain stimulation technique (TPS) which markedly differs from current brain sonication concepts by using ultrashort ultrasound pulses instead of periodic waves and long sonication trains. Expectable advantages of TPS are better skull penetration due to a dominance of lower frequencies within the TPS pulse and absence of secondary stimulation maxima which may occur due to interaction of reflections or standing waves with long train sonication. Further, brain heating^14^, ^25^ cannot occur with the current settings of system limitations. We provide multiple preclinical evidence for TPS feasibility, safety, and efficacy. In addition, our data from a first patient study and first investigation of long‐term effects with navigated ultrasound brain stimulation, indicate also clinical TPS feasibility, safety, and preliminary efficacy. TPS is clinically certified for AD therapy (CE mark).

Concerning the preclinical experiments, our results demonstrate that TPS can generate well focused brain stimulation pulses within the brain. The simulations, laboratory, and in vivo animal measurements also indicate broad safety margins according to the current state of acquired data. Even energy levels much above those used in the clinical study did not induce any hemorrhage or brain injury: the in vivo rat studies applied up to 100 pulses of 0.3 mJ mm^−2^ or 150‐fold energy levels compared to the human maximum at the same brain location and with a rat skull absorption rate of only 30% of human skulls. Follow up studies might also control for blood brain barrier opening,^23^ although our energy levels render it unlikely, that this occurred in our subjects. Efficacy of TPS for neuromodulation was tested with a sham controlled SEP study in healthy subjects. Comparable to previous studies performed with long‐train sonication,^7^, ^8^ various SEP components of the brain were increased or decreased by TPS (relative to sham stimulation). In extension, we could also demonstrate a dosing effect. First differences to the sham condition were already evident after ten TPS pulses for the component N140 which is related to conscious stimulus perception. When increasing the number of pulses up to 1000 pulses, early SEP components (P27 and N70) related to primary and secondary somatosensory processing^26^ were additionally modulated by TPS.

For initial testing of the new technology for clinical applicability, an uncontrolled multicenter AD study has been set up for judging safety and preliminary clinical efficacy. The neuropsychological evaluation provides first long‐term data for ultrasound brain stimulation. Interestingly, improvements indicate three month long‐term effects. Besides improvements in the language domain, there was particular improvement of memory performance. This was backed by the fMRI data which revealed enhanced activation and connectivity of the memory network after TPS therapy (other networks showed no significant response). It is important that these effects were achieved in an AD population already receiving optimized standard treatment indicating that TPS may be used as an independent add‐on therapy. Regarding site specific outcomes, differences between centers were small despite having used different stimulation approaches. This is probably related to the fact that in this pilot study the navigated and the non‐navigated procedure both stimulated large areas of the cortex in a population with widespread pathology. For the figural network, however, an interesting site‐specific effect was found. Figural tests showed a significant decline after three months driven by the constructional praxis results of center 1 only. Center 1 performed a navigated stimulation of the AD network instead of a global brain stimulation and did not cover all areas relevant for constructional praxis (e.g., the occipito‐parietal cortex was not treated). Therefore, a site‐specific effect seems reasonable: at center 1 the stimulated memory network was upregulated. In contrast, figural abilities related to the nonstimulated occipto‐parietal areas declined, which may be compatible with the natural course of the disease.

Regarding the precise mechanism of action, specifically how ultrasound may affect neurons and may generate neuroplastic effects, current knowledge is limited. Several principles, related to different ultrasound‐based techniques, have been proposed.^2^, ^13^, ^24^, ^27^ The likely basis are mechanical effects on cell membranes affecting mechanosensitive ion channels and generating membrane pores.^28^ As a consequence, transmitter and humoral factor concentrations may change. Increases in extracellular serotonin and dopamine levels, reduction of GABA levels, increase of brain‐derived neurotrophic factor (BDNF), glial cell line‐derived neurotrophic factor (GDNF), and vascular endothelial growth factor (VEGF) have been described.^29^, ^30^, ^31^ This may support cellular and network changes. Using ultrashort ultrasound pulses and a neuronal stem cell culture, stem cell proliferation and differentiation to neurons could be enhanced.^32^ In an AD mouse model, microglia activation with plaque reduction, clearing of Aβ into microglial lysosomes and improvements of spatial and recognition memory have been shown.^33^ Another study suggested an important role of nitric oxide synthase when improving cognitive dysfunctions in mouse models of dementia by whole brain stimulation.^18^

Concerning study limitations, the patient pilot study was performed with an uncontrolled design and therefore further sham‐controlled investigations are required to confirm the stimulation effects. However, the data contain several lines of independent evidence for clinical TPS efficacy. The long‐term course of our neuropsychological improvements clearly differs from the expected placebo responses.^34^ The dissociation between improved memory and language but worsened figural functions is also incompatible with placebo or repetition effects. In addition, the subgroup fMRI data back a specific upregulation of the memory network. For a more differentiated assessment of clinical efficacy, follow up studies should compare patient subgroups regarding disease stage, extent of antidementia therapy, comorbidities, and cognitive status. As our primary interest was to judge safety and feasibility on a broad range of patients in a realistic outpatient setting, we did not recruit a homogeneous AD group for this pilot study. TPS safety could be shown in our animal, healthy subjects, and patient data. We realize, that a definitive judgement of safety issues requires additional data from larger populations. Note however, that our animal data indicate large safety margins and more than 1500 experimental human pilot applications have already been performed over the last years without evidence for major side effects (unpublished data from the certification process). Regarding the optimal mode of TPS application, our experimental design is just an initial step. Although we made sure that energy transmission and safety issues were clarified before starting a patient intervention, future studies should clarify procedural optimizations including development of a standard brain sonication concept. Such a concept might also influence current FUS concepts since these systems might also be able to emit single short pulses and similar energy levels. Future studies might also include investigations relating local skull thickness and focal energy within the brain, although—in contrast to multichannel systems—the technical and physiological relevance for single channel transducers is reduced. In our human skull measurements the differences between human skulls concerning focus distortions and energy absorptions were minor compared to the differences between animal and human skulls. However, future studies might consider standardizations of skull thickness for the inclusion criteria. Further, a combination of brain stimulation with cognitive tasks might improve clinical outcome.^35^, ^36^

We conclude that TPS allows precise targeting of brain network areas which involves two aspects: (1) stimulation of larger cortical ROIs with well definable stimulation borders and (2) precise stimulation of smaller foci or even deep network nodes. This is a particular advantage over electrophysiological brain stimulation techniques, where focality, targeting, and stimulation of deep brain structures are generally difficult issues, especially in pathological brains.^4^ Therefore, TPS represents a promising novel brain stimulation technique with a mobile system adequate for human research and brain stimulation therapy. Results encourage broad neuroscientific application and translation of the new method to clinical therapy and randomized sham‐controlled studies.

## 4 Experimental Section

For all procedures described in this manuscript appropriate Ethical Committee approval and informed consent of all participating subjects was obtained. For all animal experiments permission was obtained from the relevant local authority “Regierungspräsidium Tübingen, Germany”. All regulations required by the CE authorities have been fulfilled for acquisition of the CE mark for TPS.

*TPS Focal Energy Transmission*: The simulations and laboratory experiments were set up to investigate the following issues: (1) Can TPS transmit energy adequately through the skull? (2) Can TPS generate a small focused sonication beam below the skull?

Detailed experimental descriptions for (1) TPS data simulations, (2) human skull and brain sample measurements as well as (3) rat skull measurements can be found in the Supporting Information.

*TPS Safety and Neuromodulatory Efficacy*: TPS safety and neuromodulatory efficacy were investigated by two experiments using anesthetized rats (85 rats in total) and one experimental series for modulation of somatosensory evoked potentials in healthy human subjects. Detailed experimental descriptions for the rat studies can be found in the Supporting Information.

*TPS Safety and Neuromodulatory Efficacy in Healthy Subjects*: Ten healthy male subjects (mean age 30.90, SD = 7.09) underwent a randomized, sham‐controlled, and single‐blind study with highly focal stimulation of the primary somatosensory representation of the right hand with fixed head‐handpiece positions (0.25 mJ mm^−2^; 4 Hz). Median nerve SEPs were recorded after sham and verum TPS blocks consisting of 10, 100, or 1000 TPS pulses in fixed order (Figure 2). Sham stimulation was achieved by blocking the ultrasound beam with a sham cap on the TPS handpiece that looked identical and produced a similar sound as the verum stimulation. SEPs were elicited by electrical stimulation of the right median nerve (intensity = individual motor threshold; frequency = 1.3 Hz, Number of pulses = 480; duration about 6:15 min). A recording electrode was positioned immediately posterior to the TPS hand piece corresponding to about CP3, a reference electrode at FPZ, and a ground electrode at the left mastoid process. Data were recorded within one session applying a sham and a TPS block (order counterbalanced between subjects) with four identical runs of SEP recordings (Figure 2B). EEG data were acquired using a DC amplifier (BrainAmp) and EEG analyses were performed with EEGLab.^37^ Data preprocessing included filtering (band‐pass filter 2–90 Hz, Notch filter 50 Hz), epoching (−100 to 600 ms around the median nerve stimulation), baseline‐correction (−100 to 0 ms), and automated artifact detection (range [−50 + 50 µV], 75 µV maximum difference within epoch). TPS effects were analyzed with a factorial design with Condition (Sham/Verum) and TPS pulse count (10/100/1000) as factors. EEG analyses were done according to Legon et al. using trial‐wise data (all valid epochs) and nonparametrical permutation statistics (1000 permutations, *p* < 0.025).^8^

*TPS Clinical Efficacy and Safety*: The multicenter clinical pilot study was designed to investigate the following issues: (1) Is TPS safe and feasible in a broad range of patients and with varying treatment durations? (2) Are there indications for preliminary effects as investigated by neuropsychological scores and fMRI data? (3) Does the mode of TPS application show relevant differences concerning issues (1) and (2)? For the latter a non‐navigated global cortical stimulation (center 2) was compared with a ROI based stimulation (center 1) with precise targeting of cortical AD network areas (see Brain Stimulation Procedure) and clearly defined stimulation borders (requiring high focality of the technique).

*TPS Clinical Efficacy and Safety—Patients*: To adequately evaluate safety and feasibility on a wide range of patients, the TPS pilot study was performed within a broad clinical setting for outpatients with memory complaints related to probable AD. Although most patients suffered from mild to moderate AD (Mini‐Mental State Examination (MMSE) value ≥18), an MMSE cutoff was not used to minimize inclusion criteria and to enable patient variability (including controlled and stable comorbidities, see Tables S2 and S3 in the Supporting Information). Relevant intracerebral pathologies unrelated to AD and independent neuropsychiatric disease (like preexisting depression) were excluded. One center in Austria (center 1 Vienna, lead) and one center in Germany (center 2 Bad Krozingen) included 20 AD patients each. Only patients receiving already optimized standard treatments were accepted and inclusion was based on clinical evaluation and external clinical MRIs. Recruitment was performed by independent neurologists with consecutive referrals to the study centers. Due to dropouts, per protocol analysis was possible for 35 patients (19 Center 1, 16 Center 2).

Common Inclusion Criteria

– Clinically stable patients with probable AD (diagnosis according to the criteria given in ICD‐10 (F00) and the NIA‐AA criteria by an expert in cognitive neurology)
– At least three months of stable antidementia therapy or no antidementia therapy necessary
– Signed written informed consent
– Age ≥18 years

Common Exclusion criteria

– Noncompliance with the protocol
– Relevant intracerebral pathology unrelated to the AD (e.g., brain tumor)
– Hemophilia or other blood clotting disorders or thrombosis
– Corticosteroid treatment within the last six weeks before first treatment
– Pregnant or breastfeeding women

*TPS Clinical Efficacy and Safety—Brain Stimulation Procedure*: Since neurodegeneration in AD brains is widespread and promising animal data for whole brain ultrasound therapy in Alzheimer's mouse models exist,^18^ non‐navigated global cortical stimulation (center 2) was compared with navigated AD network stimulation (center 1). Center 1 used ROIs that were ellipsoids defining the stimulated brain area which should precisely be targeted (requiring a highly focal technique, Figure 1C). TPS was performed with single ultrasound pressure pulses: duration about 3 µs (Figure 1C), 0.2 mJ mm^−2^ energy flux density, pulse repetition frequency 5 Hz, pulse number per therapeutic session 6000. A NEUROLITH TPS generator (Storz Medical AG, Tägerwilen, Switzerland) was utilized. The treatment comprised three sessions per week for 2–4 weeks.

(1) Detailed stimulation procedure at center 1: Individual ROIs were defined by a neurologist to target AD relevant brain areas (AD network). ROIs included the classical AD stimulation target dorsolateral prefrontal cortex and areas of the memory (including default mode) and language networks. According to an anatomical pre‐evaluation of brain size variability (in house software for gross estimation of cerebrum size), two sets of standardized ROI sizes were established and applied for either small or large patient brains. Every ROI was stimulated twice per session and most patients were stimulated for four weeks (three patients for two weeks, one for three weeks). Individual ROIs comprised: bilateral frontal cortex (dorsolateral prefrontal cortex and inferior frontal cortex extending to Broca's area, ROI volume 136/164 cm³ – 2 × 800 pulses per hemisphere), bilateral lateral parietal cortex (extending to Wernicke's area, ROI volume 122/147 cm³ – 2 × 400 pulses per hemisphere), and extended precuneus cortex (1 bilateral volume with 66/92 cm³ – 2 × 600 pulses). The goal was to distribute all pulses within the respective ROIs with a focus on the cortical tissue (Figure 1C).

(2) Stimulation procedure center 2: A nonstandardized and non‐navigated global brain stimulation approach was performed to compare different modes of TPS stimulation, for comparison see Eguchi et al.^18^ Here, the goal was to homogenously distribute the total energy of 6000 TPS pulses per session over all accessible brain areas over a treatment period of two weeks. For this, the TPS handpiece was moved along the anterior‐posterior skull axis over the whole scalp as well as in circular motions around the head without fixed trajectories.

*TPS Clinical Efficacy and Safety—Neuropsychological Evaluation*: Neuropsychological tests were performed before the stimulation (baseline), in the week after (poststim) as well as one month (one month poststim), and three months after the last stimulation session (three month poststim).

(1) CERAD: The German version of the CERAD Plus (including Trail Making Test and Phonemic word fluency) was used for testing neuropsychological functions.^16^ The CERAD is well suited for mild dementia evaluations since it does not show repetition effects with mild AD.^38^, ^39^ Additionally, the CERAD highly correlates with other global cognitive and functional scales including ADAS‐COG.^40^ Evaluations include word fluency (phonemic and categorical), naming (Boston Naming Test), encoding, recognition, and recall of verbal material (Word list), as well as constructional praxis and constructional recall (Figures Copy and Recall). The Trail‐Making‐Test was accomplishable in only about half of the patients and was thus excluded from final analyses. The CERAD raw scores were used to calculate the corrected total score (CTS,^41^ *N* = 35 complete datasets). The logistic regression score (LR,^16^ *N* = 31) and the PCA score (*N* = 30) were generated using the z‐transformed scores (corrected for age, gender, and formal education as performed by the CERAD Online analysis; norm population CERAD: *N* = 1100, phonemic word fluency: *N* = 604). The LR score weights those CERAD subtests which are particularly indicative of AD type dementia.^42^ The PCA on all CERAD subtests defined statistically independent factors that explained individual test performance with an eigenvalue >1. This approach is similar to the PCA approach by Ehrensperger et al.,^16^ but it additionally includes the phonemic word fluency test. For the PCA, the rotation method Varimax with Kaiser normalization was used (SPSS v24).

(2) Assessments of depressive symptoms: As depression is a typical comorbidity of AD, effects on depressive symptoms were monitored with the Geriatric Depression Scale (GDS, 30 complete patient datasets) and the Beck Depression Inventory (BDI, 25 complete patient datasets). As GDS and BDI values were not normally distributed according to the Kolmogorow–Smirnow‐Test, statistical evaluation was performed with the nonparametric Friedman‐Test for multiple paired variables (SPSS v24).

(3) Statistical analysis: Statistical analysis of the dependent variables (CERAD CTS, LR score, PCA factors) was done with SPSS v24 applying a Test mixed ANOVA with TIME as within‐subject factor (baseline, poststim, one month poststim, three months poststim), and CENTER (1, 2) as between‐subject factor. Spearman rank correlation analysis was used to evaluate correlations between the CERAD variables and the depression scores (GDS, BDI).

*TPS Clinical Efficacy and Safety—MRI measurements*: At center 1, all patients underwent MR investigations (3 T SIEMENS PRISMA MR with a 64‐channel head coil) including anatomical scans used for navigation via the tracking and visualization tool (*N* = 19 complete datasets). In addition, resting state scans (*N* = 18) as well as T2* and FLASH images for safety evaluations (bleedings, edema, morphology) were recorded before and after the stimulation interventions. A subgroup of nine patients underwent an additional fMRI memory task (*N* = 19).

(1) MRI sequence parameters: A T1‐weighted structural image was recorded using a MPRAGE sequence (TE/TR = 2.7/1800 ms, inversion time = 900 ms, flip angle = 9°, resolution 1 mm isotropic). For judgement of bleedings and other pathologies T2* weighted and FLASH images were recorded in every MRI session. For functional images a T2* weighted gradient‐echo‐planar imaging (EPI) sequence was used, with 38 slices aligned to AC‐PC, covering the whole brain including cerebellum (TE/TR = 30/2500 ms, flip angle = 90°, in‐plane acceleration = GRAPPA 2, field of view = 230 × 230 mm, voxel size = 1.8 × 1.8 × 3 mm, 25% gap). 98 volumes (4 min 5 s) for task fMRI and 250 Volumes (10 min 25 s) for resting state fMRI (with fixation cross) were recorded.

(2) Task fMRI: A well‐known face‐name encoding task,^19^ often used in AD studies, was applied during fMRI in a subset of the patients (*N* = 9). This task consisted of 6 runs (about 4 min each) with four activation blocks (two blocks novel face‐name associations, two blocks repeated face‐name associations, 40 s each) alternating with three rest blocks (25 s each). To promote deep encoding and to assure attention to the task, the patients should indicate by pressing a button if the name fits the face based on their subjective impression (for each face‐name pair, presented for 5 s).^19^

(3) Data analysis task fMRI: Standard preprocessing was performed by using SPM12 including realignment, coregistration, structural segmentation, normalization to MNI space and smoothing (8 mm FWHM Gaussian kernel). For first level analysis contrasts for the tasks (novel, repeated) for each session (baseline and poststim) and for each subject were calculated. The following regressors of “no interest” were used as physiological noise regressors defined with aCompCor.^43^ (1) six motion parameters derived from the realignment procedure and (2) the first three eigenvariates extracted from non gray‐matter tissue masks (cerebrospinal fluid, white matter, bone, soft tissue masks derived from the segmentation procedure). Second level analysis was done with a factorial design with task (novel, repeated) and session (baseline, poststim) as factors. The contrast of interest was defined as “novel versus repeated” for each session separately and followed by a contrast between sessions.

(4) Resting state fMRI analysis: All preprocessing procedures and analyses were performed using the CONN toolbox v17.^44^ This included default preprocessing: realignment, unwarping, slice‐time correction, structural segmentation, normalization, outlier detection (ART‐based scrubbing), and smoothing (8 mm FWHM kernel). Denoising was done using a band pass filter [0.008–0.09 Hz], removal of motion confounds (six motion parameters and their first derivatives), definition of five PCA components extracted from the cerebrospinal fluid and the white matter masks (aCompCor,^43^) and scrubbing. For first level analysis, a bivariate correlation of the corrected time series of all voxels was calculated.

(5) Network definitions for the resting state analysis: Networks investigated comprise predefined networks of the CONN‐Toolbox derived from ICA analyses of the HCP dataset (497 subjects) related to cognitive functions, a memory network (literature‐based selection of regions), and a network of the stimulated areas:

  – Default mode network (CONN)—medial prefrontal cortex, bilateral lateral parietal cortex, precuneus
  – Salience network (CONN)—anterior cingulate cortex, bilateral anterior insula, bilateral rostral prefrontal cortex, bilateral supramarginal gyrus
  – Dorsal Attention network (CONN)—bilateral frontal eye field, bilateral inferior parietal cortex
  – Fronto‐Parietal or Central Executive network (CONN)—bilateral lateral prefrontal cortex, bilateral posterior parietal cortex
  – Language network (CONN)—bilateral inferior frontal gyrus, bilateral posterior superior temporal gyrus
  – Memory network—45 bilateral hippocampus and bilateral anterior and posterior parahippocampal cortex plus the default mode network as defined above
  – Network of brain stimulation sites (Center 1)—bilateral inferior and middle frontal gyri, bilateral inferior (supramarginal and angular gyri, parietal operculum), and superior parietal cortex, precuneus

(6) Second level functional connectivity analysis: For this, paired *t*‐tests between baseline and poststim functional connectivity (FC) were calculated (correlation of the time series in the ROIs, 0.05 FDR seed‐level correction, two‐sided) on group level. The graph theoretical measure global efficiency (GE), as an estimate of the capacity of parallel information processing within a network,^46^ was calculated and compared between the baseline and the poststim session (adjacency matrix threshold: correlation coefficient = 0.35 to define connected ROIs; analysis threshold 0.05 FDR corr., paired *t*‐tests, two‐sided).

(7) Correlation of graph theoretical values with neuropsychological scores: GE values for networks showing a significant difference between sessions were extracted for all subjects and sessions and were used for correlation analysis with CERAD scores (CTS, LR, PCA factors). Data of both MR sessions entered the correlation analysis which was performed with SPSS 24 using a nonparametric Spearman rank correlation analysis as GE values were not normally distributed.

*TPS Clinical Efficacy and Safety—Patient Evaluations*: At both centers, patient reports were acquired at each visit. Center 1 used additional questionnaires to acquire further quantitative information on patients' treatment experience, including improved functionality and tolerability. These included the German SEG scale (“Skala zur Erfassung der Gedächtnisleistung” = scale for subjective evaluation of memory performance), Inventory of activities of daily living (IADL) questionnaire and a German scale for leisure activity (“Freizeitverhalten”). These questionnaires were applied at all four time points of neuropsychological testing (baseline, poststim, one month poststim, three months poststim).

In addition, after each of the treatment sessions patients evaluated their pain and pressure experience during treatment (visual analogue scales, 0 = none, 10 = very strong pain/pressure). Patients also reported on side effects with nonstandardized answers. For cognitive changes, changes of general activity, mood changes, and “change of body state” (control question) patients' answers were categorized in “improved,” “stable,” and “worsened.” To check for intracerebral pathologies, anatomical MRIs as well as T2* and FLASH images were evaluated after each MRI session.

## Conflict of Interest

The authors declare no conflict of interest.

## Author Contributions

General ideas for the experiments were contributed by E.Mar., R.B. (preclinical) and R.B., H.LB., E.Mar (clinical). Design of experiments was done by E.Mar., R.B., E.M., C.F., C.G. (preclinical) and R.B., H.LB., E.Mar., C.F., E.M., H.B., J.L. (clinical). Data collection was done by E.Mar., C.G., C.F., R.B., E.M. (preclinical) and H.LB., T.PN, H.B., E.M., M.Sch., A.A., T.A., R.R., A.W., R.B., U.R. (clinical). Analysis and interpretation of data were performed by E.Mar., E.M., C.G., R.B., E.M., C.F. (preclinical) and R.B., E.M., M.Sch., C.F., H.B., H.LB, M.H. (clinical). The manuscript was written by R.B., E.M., C.G., M. Sch., M.H. All authors contributed to editing and revising the manuscript.

## Supporting information

Supporting Information
