# Supplementary material for: Transcranial Pulse Stimulation with Ultrasound in Alzheimer's Disease—A New Navigated Focal Brain Therapy
Source: Adv Sci (Weinh). 2019 Dec 23;7(3):1902583. doi: 10.1002/advs.201902583 (PMC7001626; doi:10.1002/advs.201902583)
Supplement: Supplementary file 1 — Supporting Information [file ADVS-7-1902583-s001.pdf]

Copyright WILEY-VCH Verlag GmbH & Co. KGaA, 69469 Weinheim, Germany, 2019.

ADVANCED SCIENCE
Open Access

Supporting Information

for *Adv. Sci.,* DOI: 10.1002/advs.201902583

Transcranial Pulse Stimulation with Ultrasound in Alzheimer's Disease—A New Navigated Focal Brain Therapy

Roland Beisteiner, Eva Matt, Christina Fan, Heike Baldysiak, Marleen Schönfeld, Tabea Philippi Novak, Ahmad Amini, Tuna Aslan, Raphael Reinecke, Johann Lehrner, Alexandra Weber, Ulrike Reime, Cédric Goldenstedt, Ernst Marlinghaus, Mark Hallett, and Henning Lohse-Busch*

Supporting Information

Transcranial Pulse Stimulation with Ultrasound in Alzheimer's Disease – A New Navigated Focal Brain Therapy

Roland Beisteiner, Eva Matt, Christina Fan, Heike Baldysiak, Marleen Schönfeld, Tabea Philippi-Novak, Ahmad Amini, Tuna Aslan, Raphael Reinecke, Johann Lehrner, Alexandra Weber, Ulrike Reime, Cédric Goldenstedt, Ernst Marlinghaus, Mark Hallett, Henning Lohse-Busch*

1. Supplementary Results -– Preliminary evidence for TPS clinical efficacy and safety

1.1. Neuropsychological improvements in Alzheimer's disease

1.1.1. CERAD Corrected Total Score (CTS)

CERAD CTS score analysis (N = 35) revealed a significant within-subjects effect of TIME: $P < .0001$. The between-subjects effect of CENTER was not significant ($P = .313$). This indicates that CTS values differ between the 4 time points, but not overall between centers. Post-hoc pairwise comparisons of CTS values (Bonferroni corrected) unveil significant differences for the following: baseline < post-stim ($P_{Bonf} < .0001$), baseline < 1month post-stim ($P_{Bonf} < .0001$), baseline < 3months post-stim ($P_{Bonf} < .0001$; see **Supplementary Table S4** and Figure 3A). Furthermore, a significant interaction TIME*CENTER ($P = .003$) was found indicating that CTS differences between time points vary between the centers. A follow-up repeated measurements ANOVA for both centers separately revealed a significant main effect of TIME for both centers individually. All 3 pairwise comparisons remained significant for center 2, whereas for center 1 only the baseline < post-stim contrast reached significance.

1.1.2. CERAD Logistic Regression (LR) score

For the CERAD LR score (N = 31) a significant within-subjects effect of TIME ($P < .0001$, Greenhouse-Geisser corrected since the assumption of sphericity was violated) was found. In contrast, the between-subjects effect of CENTER was not significant ($P = .830$) indicating that LR values differ among the 4 time points but not over all between the centers. Post-hoc pairwise comparisons revealed significant differences for baseline < post-stim ($P_{Bonf} < .0001$), baseline < 1month post-stim ($P_{Bonf} < .0001$), baseline < 3months post-stim ($P_{Bonf} < .0001$), post-stim < 1month post-stim ($P_{Bonf} = .012$; Table S4, Figure 3B). As for the CTS score, a significant interaction TIME*CENTER ($P = .038$) was found. Further, the main effect of TIME was significant for both centers in a repeated measurements ANOVA for both centers separately. Again, for center 2 all 3 baseline comparisons remained significant, but for center 1 only the baseline < 1month post-stim comparison reached significance.

1.1.3. CERAD Principle Component Analysis (PCA)

Three factors achieved eigenvalues greater than 1 which means that they explained more variance than every single subtest taken alone (**Table S5**). Factor 1 (eigenvalue = 5.09, explained variance = 46.25%) displayed the highest loadings on the delayed recall and recognition of the Word List and on Savings of the Word List and the Figures and was thus named Factor MEMORY. Factor 2 (eigenvalue = 1.53, explained variance = 13.95%) was interpreted as VERBAL as its highest loadings were found for the Verbal Fluency tasks and the Word List Total. The loadings of Factor 3 (eigenvalue = 1.19, explained variance = 10.77%) were highest for the figural tasks and this factor was termed FIGURAL.

Factor 1 (MEMORY)

A mixed ANOVA with the PCA factor loadings (N = 30) showed a significant within-subjects effect of TIME: $P < .0001$ (Table S4, Figure 3C). The between-subjects effect of CENTER (P =

.606) as well as the interaction TIME*CENTER (P = .482) were not significant. Post-hoc pairwise comparisons showed significant differences for baseline < 1month post-stim (P_{Bonf} = .002) and baseline < 3months post-stim (P_{Bonf} = .002).

Factor 2 (VERBAL)

Again, the mixed ANOVA showed a significant within-subjects effect of TIME: P < .0001 (Table S4, Figure 3D). The between-subjects effect of CENTER was not significant (p = .137). Post-hoc pairwise comparisons demonstrated significant differences for baseline < post-stim (P_{Bonf} = .003), baseline < 1month post-stim (P_{Bonf} = .001), baseline < 3months post-stim (P_{Bonf} < .0001). Further, a significant interaction TIME*CENTER (P = .002) was found indicating that factor differences between time points vary between centers. The main effect of TIME was significant for center 2 only (repeated measurements ANOVA for the centers separately) and all 3 pairwise comparisons remained significant.

Factor 3 (FIGURAL)

Factor 3 revealed a significant within-subjects effect of time in the sense of a decline (P = .014; Table S4, Figure 3E). The between-subjects effect of CENTER was not significant (P = .165). Post-hoc pairwise comparisons showed a significant decline for baseline > 3month post-stim (P_{Bonf} = .007). In addition, a significant interaction TIME*CENTER (P = .015) was found demonstrating that factor differences between time points differ between centers. The main effect of TIME was significant for center 1 only and the decline for baseline > 3month post-stim remained significant (repeated measurements ANOVA for the centers separately). A further qualitative evaluation indicates that this effect is primarily due to a considerable decline of constructional praxis (Figures – Copy) of the patients of center 1.

1.1.4. Depression scores cannot explain neuropsychological improvement

For the GDS, the effect of TIME was significant (P = .005). Pairwise comparisons (Wilcoxon-Test) showed GDS improvement for baseline > 3months post-stim (P_{Bonf} = .012). For BDI, effect of TIME was also significant (P < .0001). Pairwise comparisons (Wilcoxon-Test) displayed BDI improvement for baseline > post-stim (P_{Bonf} = .012), baseline > 1month post-stim (P_{Bonf} = .006) and baseline > 3months post-stim (P_{Bonf} = .012). Importantly, there was no significant correlation between BDI / GDS scores and global CERAD scores (CTS, LR) or the PCA factors after accounting for multiple comparisons (Bonferroni correction). This indicates that CERAD improvements were not driven by changes of depressive symptoms.

1.1.5. Improvement of subjective patient performance

Results from post treatment standard scales showed significant improvements in the subjective evaluation of memory performance (SEG) over time (within-subjects effect of time: P = .027, pairwise comparisons not significant). The other standard scales did not show significant changes. In the post-treatment questionnaires, up to 20% of the patients reported subjective improvements and only 2-3% aggravations (details in **Table S6**).

2. Supplementary Experimental Section

2.1. TPS focal energy transmission

2.1.1. TPS data simulations

Temporal peak intensity fields as generated by the clinically applied TPS system have been simulated for free degassed water and two real skulls including brain tissue. The numerical models were reconstructed from the CT scans of the complete heads of two donors. The position of the TPS source in relation to the skull was recreated, according to the configuration of the experimental measurements described below. The numerical simulations were performed using Matlab

(Mathworks, USA) and the open-source k-Wave toolbox, which uses a k-space pseudo-spectral time domain solution to coupled first-order acoustic equations.[1] The simulation was limited to a volume (98x50x50 mm^3) of the head containing the expected focal area and the surroundings (Figure S2A) as extracted from the CT scans. The Hounsfield Units were converted into density and acoustic celerity using the built-in k-wave functions based on the empirical results of Schneider et al. and Mast.[2,3] Absorption coefficients of 3.57 $dB.cm^{-1}.MHz^{-1}$ and 0.58 $dB.cm^{-1}.MHz^{-1}$ were respectively assigned to bone and brain structures (Szabo 2014).[4] The region outside of the skulls was modeled as non-absorbing water (ρ = 1000 $kg.m^{-3}$, c = 1489 $m.s^{-1}$). The non-linearity parameter B/A was set to 7, corresponding to most of biological soft tissues including brain,[4,5] for the whole computational domain. The pressure source was modeled as a brass parabolic reflector (c = 4198 $m.s^{-1}$; ρ = 8470 $kg.m^{-3}$) centered on a cylindrical coil, matching dimensions those of the real device. The initial acoustic excitation was simulated as a cylindrical pressure wave uniformly distributed over the coil and modelled as a single-pulse.

2.1.2. Human skull and brain sample measurements

Single pulse pressure waves were generated by a device with the same acoustic performance as the system used for the clinical study (Storz Medical AG, Tägerwilen, Switzerland). A typical pressure pulse generated by this device, measured at the focus, is shown in Figure 1B and the experimental setup is illustrated in Figure S1. The pressure pulses were measured using a needle hydrophone (Dr. Müller Instruments, Oberursel, Germany) fixed on a two-axis sliding stage. The predefined measurement domain (50 mm along the beam axis and 40 mm along the transversal axis) was centered on the geometrical focus of the handpiece, defined as the origin of the coordinate system. The spatial transversal and axial measurement steps were kept below 1 mm and 3 mm respectively. All pressure waves were released at a drive level of 0.25 mJ/mm^2, and at a pulse repetition frequency of 2 Hz. First, a reference acoustic field measurement was performed in free water. Then, a section of human skull (roughly intermediate between bregma and lambda), with the brain

parenchyma completely removed, was placed in front of the handpiece and firmly fastened in a holder. The relative position of the handpiece to the skulls was determined from photographic acquisitions during the measurements. A 3D reconstruction of the TPS handpiece and the mounting plate of the water basin was recreated in Blender software [https://www.blender.org/]. The CT scans were segmented to create a 3D surface model of the human samples. Virtual cameras using the specifications of the Canon EOS 5D Mk II were then aligned and positioned to match the reference images. The plane of incision around the circumference of the skull was determined to create a 3D model of the skull section, which was then used to reconstruct the measurement setup. These steps allowed a discrete transformation between the CT image system and real-world geometric focus position. The CT data was transformed and interpolated to the resolution of 200um, which allowed for an easy extraction of both the 3D computational volume for the simulations described above and the image plane for the visualization of the 2D measurements. The measured fields were displayed in the corresponding slice in such a way that the origin of the coordinate system of the measurement setup, representing the geometrical focus, matches the corresponding position in the CT slice (Figure S2). A similar procedure was used for measuring the pressure drop in 10 human brain samples in vitro (brain soft tissue stabilized with a net construction but without skull, 0-7 days post mortem).

2.1.3. Rat skull measurements

For allowing judgements about differences between animal skulls and human skulls, we also performed measurements of a rat skull with the same principal technology. For this, TPS was applied with 0.1 mJ/mm^2, 0.35 mJ/mm^2 and 0.55 mJ/mm^2 and at 6 positions of the rat skull: bregma point, about 5 mm left and right from the bregma, lambda point and about 5 mm left and right from the lambda (Figure S2C). Attenuation of the pulse intensity was measured at peak pulse intensity below the skull with a needle sensor (Müller-Platte needle hydrophone, Dr. Müller Instruments, Oberursel, Germany). Pulse amplitude was measured at the TPS focus below the skull.

2.2. TPS safety investigations in anesthetized rats

Single pulse pressure waves were generated by a device equivalent to the TPS system in the clinical study in 2 rat studies (Storz Medical AG, Tägerwilen, Switzerland). TPS was applied at a fixed position over the rat skull and a constant focus in the brain under anesthesia. Study 1 used 80 male Sprague-Dawley rats treated with a maximum of 0.3 mJ/mm^2 (100 pulses) (Ameln-Mayrhofer et al., in preparation). Study 2 used 5 rats treated with a maximum of 0.2 mJ/mm^2 (8000 pulses) (Shinzato et al., in preparation).

In more detail, pulse energies in study 1 were varied and sonication was performed at a frequency of 3 Hz in groups of 10 rats each - 1 control and 7 test groups with the following settings: 0.1 mJ/mm^2, 100 pulses; 0.1 mJ/mm^2, 200 pulses; 0.1 mJ/mm^2, 400 pulses; 0.2 mJ/mm^2, 100 pulses; 0.2 mJ/mm^2, 200 pulses; 0.2 mJ/mm^2, 400 pulses; 0.3 mJ/mm^2, 100 pulses. For safety evaluations, brain preparations (80 rats) and histological investigations (16 rats) were performed to investigate for possible intracerebral bleeding and tissue damage as primary outcomes. Outside the safety context of this study, animal behavior was also analyzed. Rats were held in groups in Makrolon-IV cages at fixed climatization and 12h:12h light-darkness cycles. For anesthesia isoflurane 1-2% and fentanyl 5µg/kg or Butorphanol 3,3 mg/kg were used. Analgesia was required for controlled experimental conditions and a stable brain stimulation focus. For postsurgical analgesia carprofen was used. For post treatment brain preparations, animals were decapitated. Study 2 used a similar setting and evaluated the sonication effects via in vivo MRI (see Figure S3). Total energy dose in 5 rats was varied between 400, 4000 and 8000 pulses (at 0.2 mJ/mm^2) corresponding to 15-, 150- and 300-fold energy levels relative to the human dose allowed with the certified TPS system.

3. Supplementary References

[1] B. E. Treeby, B. T. Cox, *J. Biomed. Opt.* **2010**, *15*, 021314.

[2] U. Schneider, E. Pedroni, A. Lomax, *Phys. Med. Biol*. **1996**, *41*, 111.

[3] T. D. Mast, *Acoust. Res. Lett. Online* **2000**, *1*, 37.

[4] T.L. Szabo, *Diagnostic ultrasound imaging*, Academic Press, **2014**.

[5] M. F. Hamilton, D. T. Blackstock, *Nonlinear acoustics*, Academic press, San Diego, CA **1998**.

4. Supplementary Figures

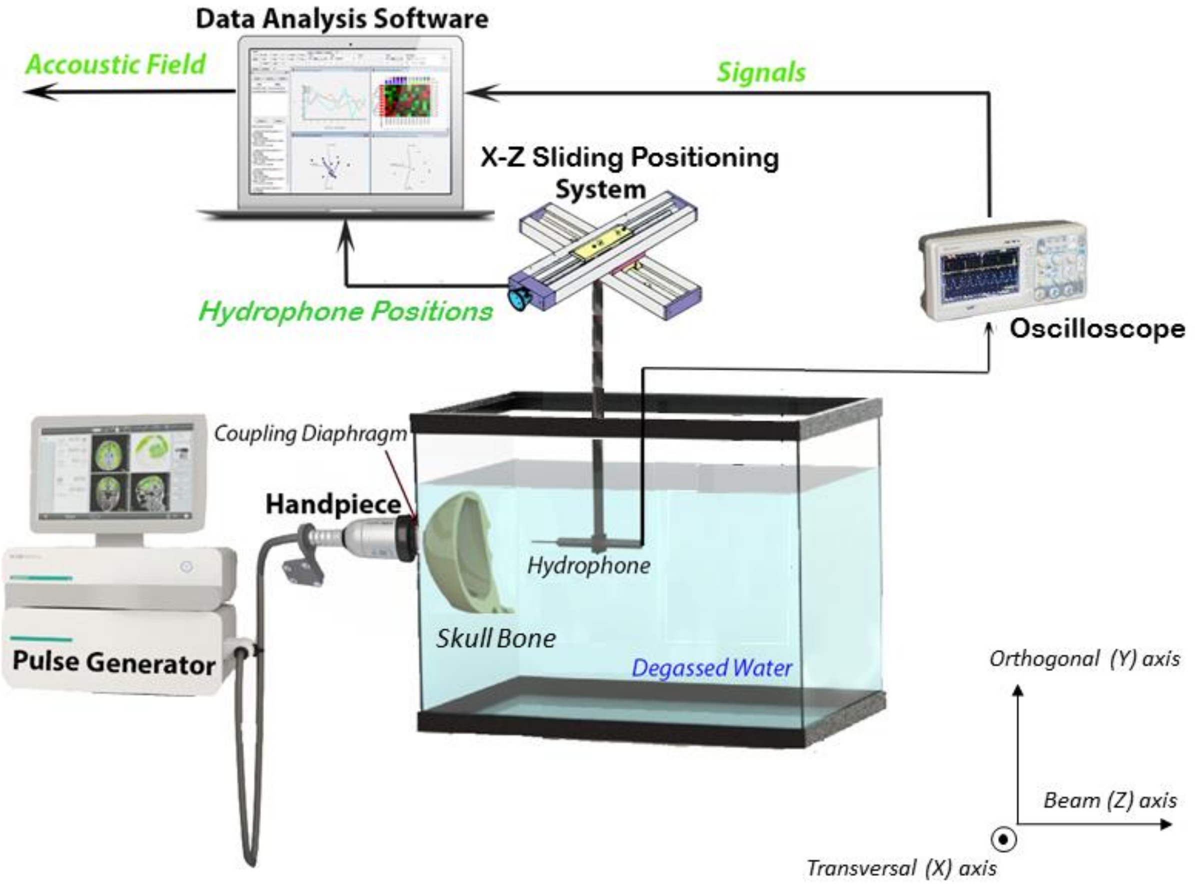

Figure S1. Experimental setup for the skull and brain sample measurements. The TPS handpiece was fixed on the side of a basin filled with degassed water. Test specimens (e.g. human skulls, rat skulls, brain specimens) were fixed directly in front of the handpiece. For brain specimen fixation a net was used. Specimen related pulse attenuations were recorded by the Hydrophone with reference to free water results (compare figure S2). The Hydrophone can be moved in 3D.

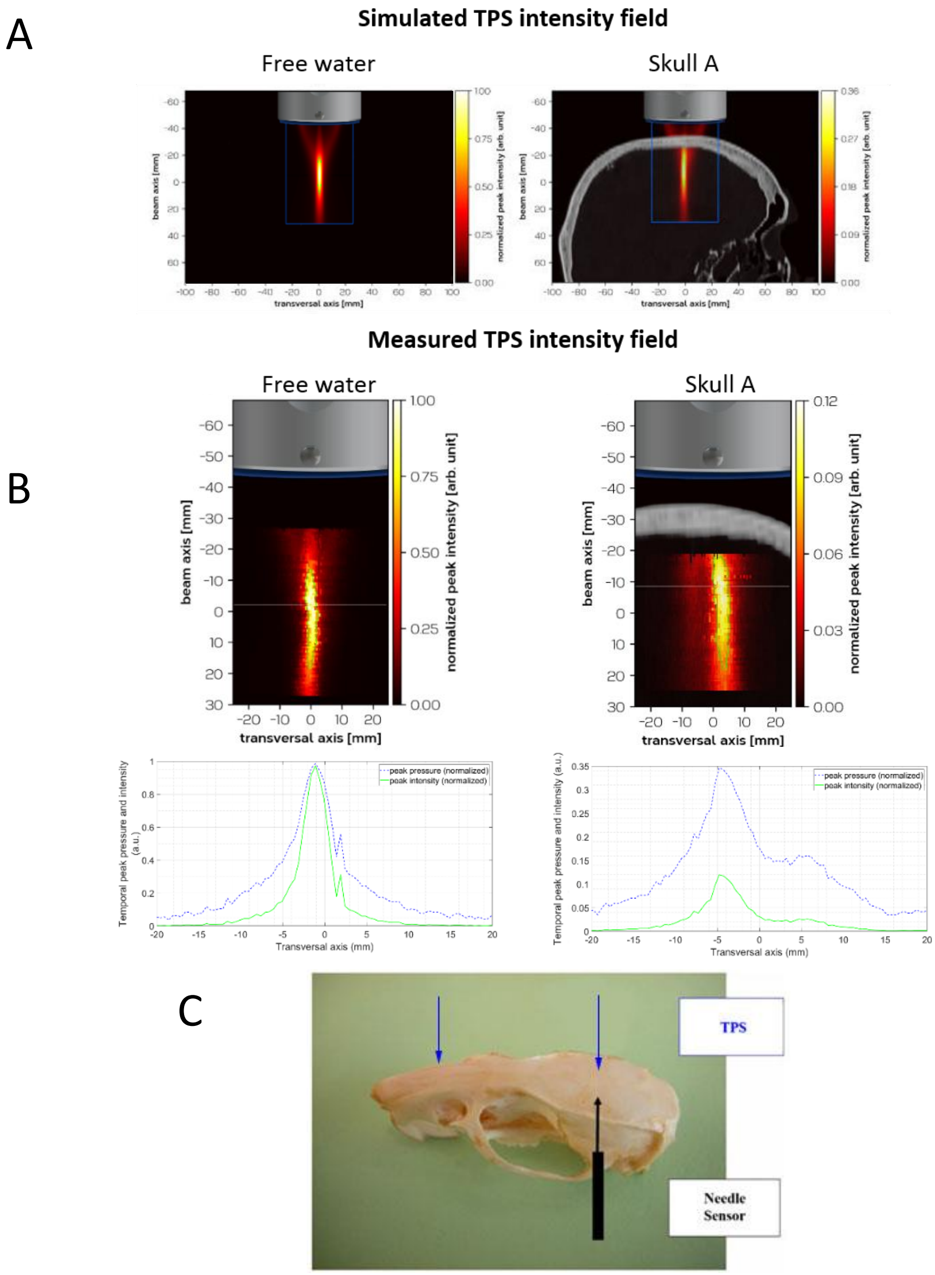

Figure S2. Simulated and measured TPS intensity field. All skull intensity and pressure values are given relative to the free water measurements. A) Simulated temporal-peak intensity field for free water (left) and for skull A (right). The blue lines indicate the limits of the computational domain. Note skull attenuation of about 65% as indicated by the color bar. B) Measured temporal-peak intensities and pressures for free water (left) and for skull A (right). Upper panel: the white line indicates a shift of the spatiotemporal peak (beam axis maximum). Lower panel: comparison of measured peak pressure and intensity distributions. The data show the focality of the pulse (= lateral spatial resolution) in the transversal plane measured at beam axis maximum. Note that measured skull attenuation is even stronger (about 85%) than expected from the simulations. C) Rat skull measurements. The blue arrows indicate that various TPS pulses were measured. On average, attenuation was only about 29%. This illustrates the importance of comparative measurements when trying to translate animal data from new ultrasound techniques to human applications.

A

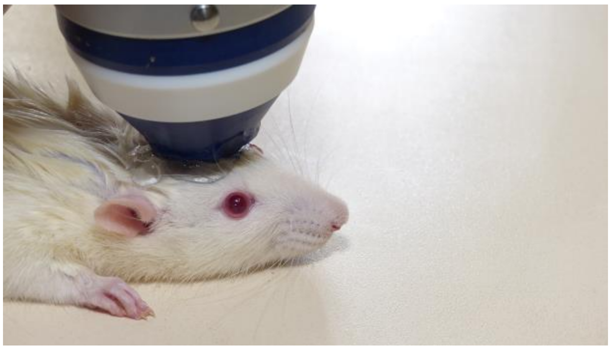

B

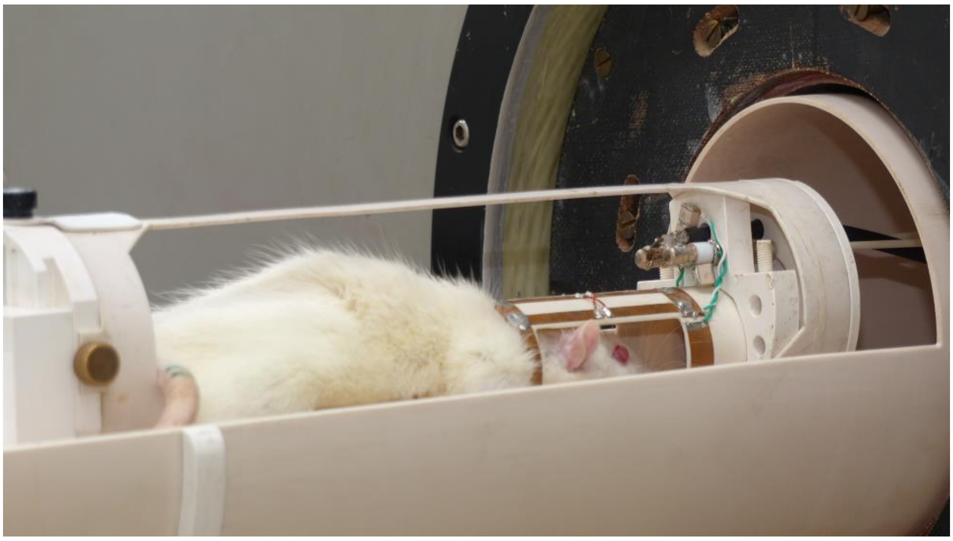

C

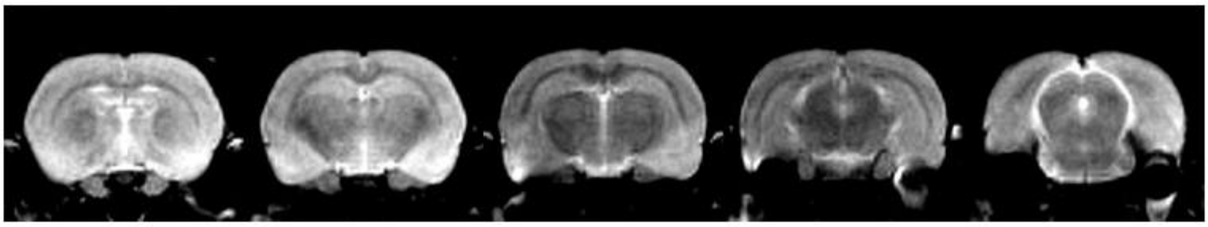

D

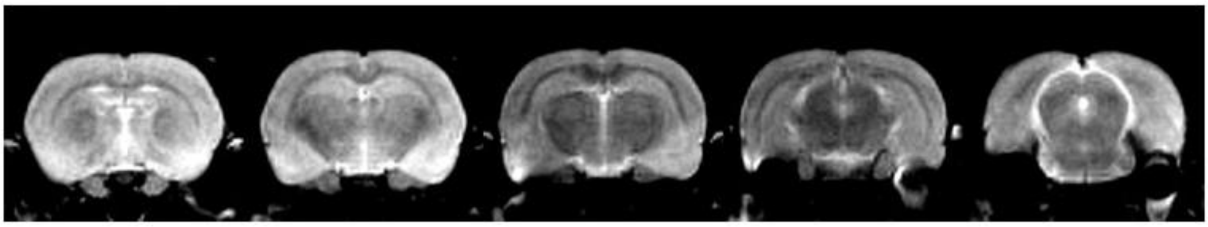

Figure S3. Evidence for TPS safety. A) Experimental setting for focal TPS stimulation and B) post stimulation MRI in rats. C) MRI results of all 5 rats after sonication with 15-fold energy levels compared to the maximum human dosis allowed. D) MRI results of all 5 rats after sonication with 150-fold energy levels compared to the maximum human dosis allowed. No brain damage is visible in C) or D) indicating high safety margins for TPS.

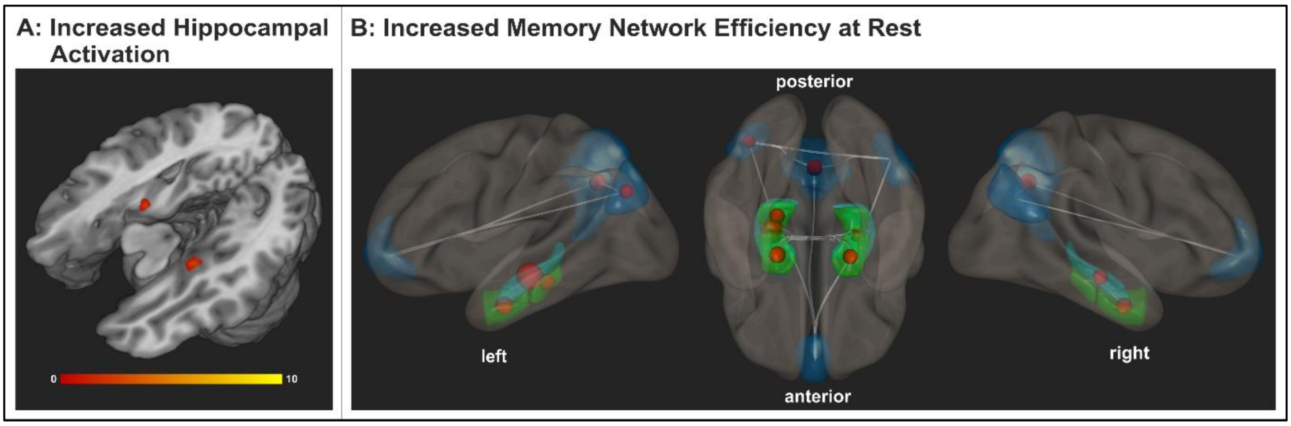

Figure S4. Increased functional activation and connectivity in the memory network after TPS brain stimulation. A) Functional activation in hippocampal areas during a face-name encoding task increased after TPS intervention compared to the baseline measurement. B) The global efficiency (GE) of the memory network (blue: default mode network, turquoise: hippocampus, green: parahippocampal areas) significantly increased after TPS in bilateral (para-) hippocampal and parietal areas (sphere size weighted according to the statistical difference between the pre and post intervention scans). Results indicate that TPS improved the function of memory areas and thereby patients' capability of mnestic information processing.

5. Supplementary Tables

Table S1. Correlation between the resting state global efficiency values in the memory network and its nodes with the neuropsychological test scores.

Global Efficiency		CTS (N = 18)	LR (N = 16)	Factor 1 – Memory (N = 16)	Factor 2 – Verbal (N = 16)	Factor 3 – Figural (N = 16)
**Memory network**	rho	**,525****	**,550****	**,450****	,357*	0,095
	p	.0010	.0011	.0097	.0446	.6043
**Parahippocampal Gyrus, anterior division R**	rho	,494*	,466*	.281	.319	.259
	p	.0022	.0071	.1190	.0756	.1515
**Parahippocampal Gyrus, anterior division L**	rho	.287	.321	.193	.284	.158
	p	.0898	.0734	.2908	.1151	.3891
**Parahippocampal Gyrus, posterior division R**	rho	.233	.225	.313	.039	-.046
	p	.1707	.2158	.0815	.8336	.8023
**Parahippocampal Gyrus, posterior division L**	rho	,386*	,475*	,503*	.221	.029
	p	.0201	.0060	.0034	.2240	.8731
**Hippocampus R**	rho	**,643****	,546*	,351*	,409*	.107
	p	.0000	.0012	.0491	.0201	.5615
**Hippocampus L**	rho	,491*	**,572****	.349	,430*	-.011
	p	.0023	.0006	.0503	.0141	.9513
**Medial Prefrontal Cortex**	rho	.292	.243	.136	.186	-.163
	p	.0836	.1804	.4594	.3088	.3724
**Lateral parietal Cortex L**	rho	**,551****	,484*	,423*	,355*	.037
	p	.0005	.0051	.0159	.0461	.8391
**Lateral parietal Cortex R**	rho	**,543****	,522*	.345	,429*	-.036
	p	.0006	.0022	.0532	.0144	.8433
**Precuneus Cortex**	rho	,512*	,448*	,381*	.335	-.024
	p	.0014	.0101	.0315	.0612	.8947

*Significant correlations without correction for multiple comparisons (α = 0.05, two-sided, Spearman rank correlation analyses); **Significant correlations with correction for multiple comparisons (Bonferroni-adjusted α = 0.01 on network level, adjusted α = 0.001 on ROI level, two-sided, Spearman rank correlation analyses). CTS = CERAD Corrected Total Score, LR = Logistic regression score, L = Left, R = Right.

Table S2. Demographic details and clinical baseline characteristics of the patient groups

		**Both Centers**<br>N = 35<br>(20 female)	**Center 1**<br>N= 19<br>(12 female)	**Center 2**<br>N = 16<br>(8 female)	**Difference between Centers** [a]
**Age**	Mean (SD)	70.37 (8.57)	68.68 (9.49)	72.38 (7.11)	*P* = .209
	Range	[51-84]	[51-84]	[58-84]	
**Education**	Mean (SD)	11.60 (3.60)	10.00 (2.00)	13.50 (4.18)	*P* = .007
	Range	[7 - 20]	[7 - 14]	[8 - 20]	
**Baseline MMSE**	Mean (SD)	20.97 (5.86)	19.53 (6.92)	22.69 (3.81)	*P* = .113
	Range	[3 - 30]	[3 - 30]	[17 - 29]	
**Baseline BDI**	N	29	14	15	
	Mean (SD)	5.62 (5.34)	7.36 (5.09)	4.00 (5.21)	*P* = .063
	Range	[0 - 18]	[0 - 16]	[0 - 18]	
**Baseline GDS**	N	33	18	15	
	Mean (SD)	3.00 (2.47)	3.50 (2.50)	2.40 (2.38)	*P* = .145
	Range	[0 - 10]	[0 - 9]	[0 - 10]	

[a] Differences between centers regarding demographic and clinical characteristics were tested using unpaired T-tests (two-sided, $\alpha = 0.05$) for normally distributed variables (age, baseline MMSE) and non-parametric Mann-Whitney-tests (two-sided, $\alpha = 0.05$) for education, baseline Beck Depression Inventory score (BDI), and baseline Geriatric Depression Score (GDS). SD = Standard deviation, MMSE = Mini-Mental State Examination

Table S3. Individual patient characteristics for Center 1 (C1) and Center 2 (C2)

ID	Age	Gender	Education	Baseline MMSE	Comorbidities
C1_01	64	female	11	3	Suspected epilepsy, tendency to collapse, hyperlipidemia, osteoporosis
C1_02	68	female	8	12	Hypertension, hypertensive encephalopathy
C1_03	72	female	11	23	Vitamin B12 deficiency
C1_04	71	male	11	14	Parkinsonian syndrome, depressive adjustment disorder, hypertension
C1_05	75	male	9	18	AD associated depression
C1_06	82	female	7	20	None
C1_07	81	female	7	17	None
C1_08	76	female	12	18	None
C1_09	56	male	9	26	AD associated depression, panic disorder, adjustment disorder, gastritis, hypertension
C1_10	67	male	14	26	Recurrent AD associated depression, vascular MAP, arachnoid cyst
C1_11	60	female	8	24	Panic attacks
C1_12	84	female	8	24	None
C1_13	61	male	12	25	None
C1_14	61	female	10	6	None
C1_15	75	female	8	21	Vascular encephalopathy, hypertension, hypercholesterolemia, AD associated depression
C1_16	76	male	12	25	Vitamin D deficiency, positive borrelia-IgG, cerebral microbleeds
C1_17	69	female	11	30	AD associated depression
C1_18	56	female	10	19	None
C1_19	51	male	12	20	AD associated depression
C2_01	71	female	13	17	Hypertension, AD associated depression, hypothyroidism
C2_02	70	male	11	21	Hypertension, diabetes II

C2_03	74	male	18	24	Coronary heart disease, hypertension, hypercholesterolemia, indigestion
C2_04	78	male	20	28	Spinal canal stenosis L4/L5, arterial hypertension, indigestion
C2_05	66	male	14	23	Paroxysmal symptomatic atrial fibrillation
C2_06	84	female	8	20	Arterial hypertension, polyneuropathy, struma nodosa, cardiac decompensation
C2_07	58	female	15	19	None
C2_09	70	female	8	23	Arterial hypertension, hypothyroidism (euthyreote struma)
C2_10	78	male	14	18	Hypertension, benign hallucinations
C2_11	79	female	8	22	Hypertension, hypothyroidism
C2_12	76	female	15	20	Hypertension, hyperuricemia
C2_13	59	male	20	29	None
C2_14	72	female	9	22	Hypertension, hypercholesterolemia
C2_15	80	male	12	27	Diabetes II, hypertension, hypothyroidism
C2_16	71	female	19	29	Intermittent spinal complaints
C2_17	72	male	12	21	Coronary heart disease, peripheral arterial occlusive disease (legs)

Table S4. Results of the mixed ANOVA for the combined dataset with post-hoc comparisons between time points

	N	Factor	F	p	η_p^{2} [a)]	Sign. Contrasts (P)[b)]
**CTS**	35	Time	$F_{(3,99)} = 18.304$	.000	.357	1 – 2 (.000)<br>1 – 3 (.000)<br>1 – 4 (.000)
		Center	$F_{(1,33)} = 1.051$	.313	.031	
		Time*Center	$F_{(3,99)} = 4.892$	.003	.129	
**LR**	31	Time	$F_{(2,69)} = 20.129$ (GG)	.000	.410	1 – 2 (.000)<br>1 – 3 (.000)<br>1 – 4 (.000)<br>2 – 3 (.012)
		Center	$F_{(1,29)} = .047$	.830	.002	
		Time*Center	$F_{(2,69)} = 3.220$ (GG)	.038	.100	
**Factor 1 (Memory)**	30	Time	$F_{(3,84)} = 7.050$	.000	.201	1 – 3 (.002)<br>1 – 4 (.002)
		Center	$F_{(1,28)} = .272$	.606	.010	
		Time*Center	$F_{(3,84)} = .828$	.482	.029	
**Factor 2 (Verbal)**	30	Time	$F_{(3,84)} = 12.433$	.000	.307	1 – 2 (.003)<br>1 – 3 (.001)<br>1 – 4 (.000)
		Center	$F_{(1,28)} = 2.339$	.137	.077	
		Time*Center	$F_{(3,84)} = 5.351$	.002	.160	
**Factor 3 (Figural)**	30	Time	$F_{(3,84)} = 3.739$	.014	.118	1 – 4 (.007)
		Center	$F_{(1,28)} = 2.033$	.165	.068	
		Time*Center	$F_{(3,84)} = 3.708$	.015	.117	

[a)] Partial eta squared as an estimate for the effect size; [b)] Post-hoc comparisons between time points (1 = baseline, 2 = post-stim, 3 = 1month post-stim, 4 = 3months post-stim) were corrected for multiple comparisons using Bonferroni correction. CTS = CERAD Corrected Total Score, LR = Logistic regression score, GG = Greenhouse-Geisser corrected.

Table S5: PCA factor loadings of the CERAD subtests

	**Factors[a)]**		
	**1**	**2**	**3**
**CERAD variables**	**Memory**	**Verbal**	**Figural**
Animal Fluency	.118	**.872**	.306
Boston Naming Test	.312	**.478**	.146
Word List – Total	.524	**.702**	.199
Word List – Delayed Recall	**.834**	.395	.159
Word List – Intrusions	**.579**	.204	.004
Word List – Savings	**.819**	-.070	.073
Word List – Recognition	**.649**	.482	.004
Figures – Copy	.005	.121	**.922**
Figures – Delayed Recall	.583	.118	**.720**
Figures – Savings	**.734**	.172	.268
Phonemic Fluency	.038	**.895**	-.113

a) Factors with an eigenvalue > 1 derived from a principle component analysis (PCA) with the rotation method Varimax with Kaiser normalization. Factors were interpreted as "Memory" (Factor 1), "Verbal functions" (Factor 2), and "Figural functions (Factor 3) according to highest loadings of the CERAD variables on the three factors (bold).

Table S6. Results from post treatment patient questionnaires

	Worsened *in %*	Stable *in %*	Improved *in %*
Cognition	2	77	20
General activity	3	82	15
Mood	3	71	26
Body state [a)]	2	93	5

a) Questions regarding the body state represent a control item as general somatic changes were not assumed to be associated with the TPS intervention. Post treatment questionnaires were acquired at Center 1 only (N = 19).
